# Methyl Metabolism and the Clock: An Ancient Story With New Perspectives

**DOI:** 10.1177/07487304221083507

**Published:** 2022-04-05

**Authors:** Jean-Michel Fustin

**Affiliations:** Centre for Biological Timing, The University of Manchester, Manchester, UK

**Keywords:** methylation, circadian, evolution, folic acid, adenosylmethionine, SAM, prebiotic

## Abstract

Methylation, that is, the transfer or synthesis of a –CH_3_ group onto a target molecule, is a pervasive biochemical modification found in organisms from bacteria to humans. In mammals, a complex metabolic pathway powered by the essential nutrients vitamin B9 and B12, methionine and choline, synthesizes *S*-adenosylmethionine, the methyl donor in the methylation of nucleic acids, proteins, fatty acids, and small molecules by over 200 substrate-specific methyltransferases described so far in humans. Methylations not only play a key role in scenarios for the origin and evolution of life, but they remain essential for the development and physiology of organisms alive today, and methylation deficiencies contribute to the etiology of many pathologies. The methylation of histones and DNA is important for circadian rhythms in many organisms, and global inhibition of methyl metabolism similarly affects biological rhythms in prokaryotes and eukaryotes. These observations, together with various pieces of evidence scattered in the literature on circadian gene expression and metabolism, indicate a close mutual interdependence between biological rhythms and methyl metabolism that may originate from prebiotic chemistry. This perspective first proposes an abiogenetic scenario for rhythmic methylations and then outlines mammalian methyl metabolism, before reanalyzing previously published data to draw a tentative map of its profound connections with the circadian clock.

## Evolution

### In the Beginning There Was. . . Chemistry

The characteristics of life, that is, responsiveness to the environment, growth, reproduction, adaptation, and homeostasis, are all found at the level of one single cell. Cellular metabolism likely originates from chemical reactions occurring on the prebiotic earth, when eobionts were evolving in an environment of countless chemical possibilities. Indeed, when bioenergetic processes are compared between extant organisms at the cellular level, the most diverse of these processes are seen among prokaryotes (Kluyver and van Niel, 1956).

### Amino Acids and Central Carbon Metabolism

One of the most famous experiments seeking to explain the origin of organic molecules now essential for life is the 1953 Miller experiment (Miller, 1953) and its later iteration (Van Trump and Miller, 1972). In a simulated primitive atmosphere, spark discharges were shown to catalyze the synthesis of the amino acids glycine, alanine, aspartic acid and methionine. Re-analysis of Miller’s archived samples with state-of-the-art analytical techniques revealed that more than 20 amino acids were abiotically synthesized (Johnson et al., 2008; Parker et al., 2011a, 2011b, 2011c). More recently, others have confirmed synthesis of amino acids in prebiotic conditions (Mompean et al., 2019; Wollrab et al., 2016; Cooper et al., 2017).

### A Step Toward Biological Methylation: Sulfur-Containing Amino Acids

Among the amino acids synthesized abiotically by a spark discharge in a reducing atmosphere containing H_2_S are the sulfur-containing amino acids methionine, homocysteine, and most likely cysteine (Parker et al., 2011c). The synthesis of methionine under an alternative sulfur-containing simulated prebiotic condition was independently reported (Steinman et al., 1968). In an evolutionary context these are especially important, because among all elements used to build amino acids (*i.e.* O, N, C, H, S) sulfur, being the most electronegative, gives amino acids different properties and makes the methyl of methionine more readily attacked by a nucleophilic molecule, essentially resulting in its methylation, with the conversion of methionine to homocysteine. Thus, methionine and homocysteine, which constitute half of the methyl cycle, may have been present on the prebiotic earth. Moreover, methionine has been shown to spontaneously react with ATP or adenosine to yield S-adenosylmethionine (SAM), the methyl donor co-substrate used by methyltransferases today (Laurino and Tawfik, 2017). Since (deoxy)ribonucleotides and (deoxy)ribonucleosides, including adenosine, can also be synthesized abiotically under conditions likely to have been prevalent on the prebiotic earth (Xu et al., 2017, 2018, 2019, 2020; Patel et al., 2015; Powner et al., 2009; Xu et al., 2021), methylation reactions using SAM may thus have occurred before the development of life. But spontaneous methylation reactions may have occurred even before methionine or SAM.

### Daily Methylation Rhythms May Have Occurred on the Prebiotic Earth

Abiotic methylation of simple amines such as ethanolamine and glycine, both products of the Miller experiment (Parker et al., 2011c), can occur in prebiotic conditions with formaldehyde (Waddell et al., 2000), a legitimate prebiotic alkylating agent readily synthesized by photolysis of water vapor with carbon monoxide (Bar-Nun and Hartman, 1978). The fact that formaldehyde can be synthesized by *photolysis* of water vapor with carbon monoxide (Bar-Nun and Hartman, 1978) is meaningful since it indicates that prebiotic methylation reactions may have occurred more frequently during the day.

Formaldehyde is highly reactive, and has been hypothesized to be the only prebiotic 1-carbon molecule able to generate more complex organic molecule, including amino acids and sugars (Weber, 2002). The main source of formaldehyde was likely gas phase photochemical synthesis in the atmosphere followed by transport into bodies of water by rain (Pinto et al., 1980; Cleaves, 2008). In aqueous solution, subsequent photochemical transformations of formaldehyde occur: oligomerization, and reactions with amines, sulfur species and HCN (Cleaves, 2008). By converting formaldehyde into non-volatile species, these reactions increase the concentration of formaldehyde. Other geochemical mechanisms, including eutectic freezing, may further increase the local concentration of formaldehyde, enabling more demanding reactions (Cleaves, 2008). Together this suggests that, during the day the amount of formaldehyde and dependent methylations would progressively increase. At night, the reactivity of formaldehyde, together with the absence of sunlight, would lead to its progressive decline.

In time, increased methylation reactions during the day may have been a way by which eobionts “sensed” daylight, and later developed strategies to avoid or expose themselves to this increased “methylation potential” according to their chemical needs. Interestingly, formaldehyde remains to this day an intermediate in some methyltransferase reactions (Meller et al., 1975; Stover and Schirch, 1990).

It is thus likely methylations occurred in the prebiotic day and imprinted their influence on abiogenesis, and cellular metabolism may have co-opted methylations as an endogenous regulatory mechanism. Now, most methyltransferases use SAM as a co-substrate, transferring the methyl moiety of SAM to their specific target substrate: nucleic acids, proteins, membrane components, hormones, xenobiotics and small molecules. It is not surprising, therefore, that SAM is the second-most used enzyme substrate (Cantoni, 1975), after ATP. If methylation reactions were more frequent during the day on the prebiotic earth, influencing the development of life, an intimate link between circadian rhythms (biological rhythms ticking with a period of a day) and methyl metabolism should be found in organisms alive today. Keeping this in mind, we first provide an overview of the methyl metabolism and its regulation in mammals.

## Methyl Metabolism Today

### Vitamin B9: Substrate For *de Novo* Methyl Synthesis

**Figure 1.**
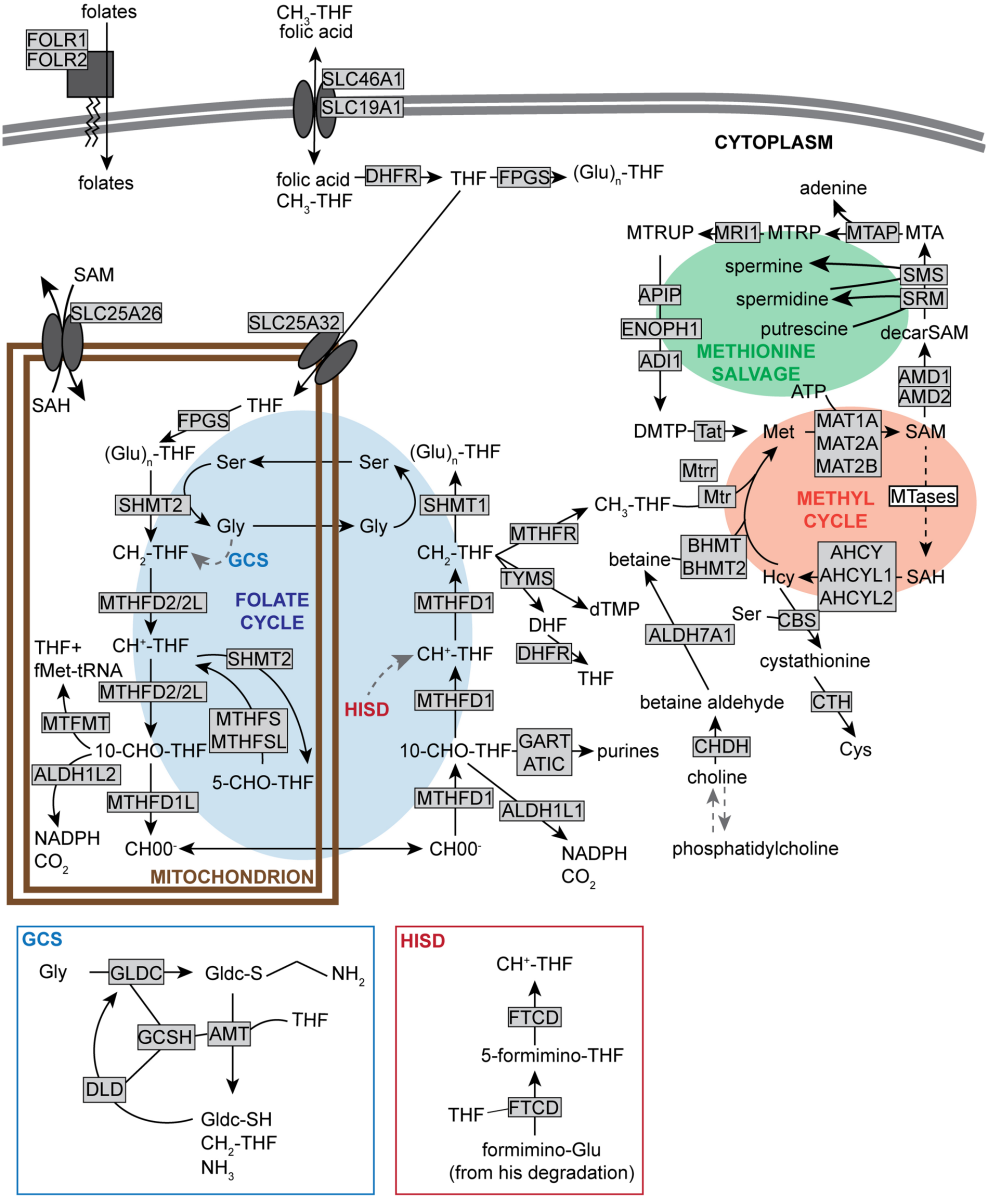
of 1-carbon (folate and methyl) metabolism. Enzymes are shown in grayed rectangle, with the exception of SAM-dependent methyltransferases, collectively represented as “MTases” in a white rectangle. Enzymes using or generating folates and/or methyl groups, as well as enzymes directly dependent on the metabolites of the methyl cycle are represented. GCS is the glycine cleavage system; through its sulfur (S), GLDC forms a bound intermediate with glycine, further cleaved into CH_2_-THF and NH_3_ by the activity of the other enzymes in the complex, with regenerated GLDC(-SH). HISD is the histidine degradation pathway, ultimately leading to the degradation of formimino-glutamate (Glu) to CH ^+^-THF mediated by FTCD. The information about enzymes mediating each step was obtained from the KEGG. Based on published reviews and pathways from public databases (Tibbetts and Appling, 2010; Clare et al., 2019; Kanehisa, 2019; Kanehisa et al., 2019; Kanehisa and Goto, 2000). Abbreviations: SAM = S-adenosylmethionine; GCS = glycine cleavage system; GLDC = Glycine decarboxylase; CH_2_-THF = 5,10-methylenetetrahydrofolate; GLDC = Glycine decarboxylase; CH ^+^-THF = 5,10-methenyltetrahydrofolate; FTCD = Formimidoyltransferase cyclodeaminase; KEGG = Kyoto Encyclopedia of Genes and Genomes; DHFR = Dihydrofolate reductase; FPGS = folylpolyglutamate synthetase; (Glu)n-THF = polyglutamylated-THF; MTRUP = S-Methyl-5-thio-D-ribulose 1-phosphate; MTRP = S-Methyl-5-thio-D-ribose 1-phosphate; Mtap = S-methyl-5'-thioadenosine phosphorylase; MTA = methylthioadenosine; Apip = Apoptotic protease activating factor 1 (APAF1)-interacting protein; Enoph1 = Enolase-phosphatase 1; Sms = Spermine synthase; Srm = Spermidine synthase; fMet-tRNA = N-formylmethionine-tRNA; MTFMT = methionyl-tRNA formyltransferase; 10-CHO-THF = 10-formyltetrahydrofolate; MTHFR = Methylenetetrahydrofolate reductase; TYMS = Thymidylate synthase; DHF = dihydrofolate; GART = phosphoribosylglycinamide formyltransferase, phosphoribosylglycinamide synthetase, phosphoribosylaminoimidazole synthetase; ATIC = 5-aminoimidazole-4-carboxamide ribonucleotide formyltransferase/IMP cyclohydrolase; MTR = Methionine synthase; BHMT = Betaine-homocysteine S-methyltransferase; ALDH7A1 = aldehyde dehydrogenase 7 family member A1; Cth = Cystathionine gamma-lyase; CHDH = choline dehydrogenase; Gly = glycine; GCSH = glycine cleavage system protein H; AMT = aminomethyltransferase; DLD = dihydrolipoamide dehydrogenase; SH = thiol; HISD = histidine degradation pathway; THF = tetrahydrofolate; SAH = S-adenosylhomocysteine; MTHFS = Methenyltetrahydrofolate Synthetase; MTHFSL = 5-formyltetrahydrofolate cyclo-ligase; 5-CHO-THF = 5-formyl-THF; dTMP = deoxythymidine monophosphate; DFHR = dehydroflate reductase; ATP = adenosine triphosphate; CBS = Cystathionine beta synthase.

The readers are invited to consult excellent reviews on methyl and folate metabolism (Tibbetts and Appling, 2010; Ducker and Rabinowitz, 2017), as only a brief overview necessary to understand the next section will be given here. The methyl of methionine mainly originates from a folate cofactor (vitamin B9) at the end of a chain of reduction (the folate cycle, Figure 1). In the folate cycle, a formate (HCOO^−^) of mitochondrial origin is added to tetrahydrofolate in the cytoplasm, forming 10-formyltetrahydrofolate (10-CHO-THF), further reduced to 5,10-methenyltetrahydrofolate (CH ^+^-THF) then 5,10-methylenetetrahydrofolate (CH_2_-THF) by the same trifunctional enzyme *methylenetetrahydrofolate dehydrogenase, cyclohydrolase, and formyltetrahydrofolate synthetase 1* (MTHFD1). Another enzyme, *methylenetetrahydrofolate reductase* (MTHFR), irreversibly synthesizes 5-methyltetrahydrofolate (CH_3_-THF) from CH_2_-THF, and *methionine synthase* (MTR) uses CH_3_-THF as a co-substrate to methylate homocysteine to methionine in a reaction that also requires its cofactor cobalamin (vitamin B12). Methionine and ATP are then converted to SAM by *methionine adenosyltransferases*, with MAT1A being expressed mainly in the liver, while MAT2A and MAT2B are ubiquitous (Shimizu-Saito et al., 1997; Ramani and Lu, 2017) (Figure 1). Collectively, folate and methyl metabolism are conventionally grouped together under the term “C1 metabolism.”

Folate metabolism is a cycle because the 1-carbon of formate leaving the mitochondria originates from serine entering the mitochondrial folate arc that is a reversal of the reactions explained above (Figure 1). The reason why cytoplasmic folate metabolism requires mitochondrial formate originates in the differential electrochemical potential between mitochondrial NADH and cytosolic NADPH, with the high cytosolic NADPH/NADP ^+^ ratio favoring the flux from formate to serine (Yang and MacKenzie, 1993). Serine is not the only amino acid used as a source of 1-carbon units, however. The glycine cleavage system (GCS) catabolizes excess glycine and converts its α-carbon into CH_2_-THF, and the histidine degradation pathway leads to CH ^+^-THF synthesis (Beaudet and Mackenzie, 1976; Kohls et al., 2000) (Figure 1).

Folates are also used as direct cofactors for several very ancient enzymes catalyzing 1-carbon transfers in the metabolism of nucleotides: TYMS catalyzes the methylation of dUMP to dTMP using CH_2_-THF (Mishanina et al., 2012); GART and ATIC use 10-CHO-THF to progressively assemble the purine base of inosine monophosphate (Nelson et al., 2017). Moreover, in bacteria and mitochondria the initiation of translation is coupled to the folate cycle via *methionyl-tRNA formyltransferase* (MTFMT), synthesizing the formylmethionine-tRNA that is essential for the initiation of proteins (Minton et al., 2018; Morscher et al., 2018). These enzymatic reactions are all shown in Figure 1.

### Transport and Sequestration of Folates

Entry of folate into the cell is controlled by the folate transporter SLC19A1, and into the mitochondrial matrix by SLC25A32. Another transporter, SlC46A1, transport folates across membranes in acidic environment. Finally, a reduced folate receptor, *Folr*, with two tissue- and developmental stage-specific isoforms in mice and three in humans, transport CH_3_-THF into the cell (Clare et al., 2019; Tibbetts and Appling, 2010) (Figure 1). Dietary folic acid, or dihydrofolate (DHF) generated by the reaction catalyzed by TYMS, need to be reduced to THF by *dihydrofolate reductase* (DHFR) to re-enter the folate cycle. Finally, to improve folate retention in the cell and its use as a cofactor, a poly-γ-glutamate tail is added to the to the *p*-aminobenzoic acid moiety of THF, a reaction catalyzed by *folylpolyglutamate synthetase* (FPGS) (Tibbetts and Appling, 2010) (Figure 1).

### Salvage of Methyl Groups From Betaine

During vertebrate evolution, the methyl cycle in adult animals became not completely reliant on folates. An enzyme called *Betaine-homocysteine S-methyltransferase* (BHMT) can re-methylate homocysteine using one of betaine’s three methyl groups (betaine is also known as *N, N*, -trimethylglycine), but only in specific tissues such as the liver and kidneys in human (Sunden et al., 1997). Unlike the *de novo* methyl of CH_3_-THF, however, the three methyl groups of endogenous betaine originate from the sequential SAM-dependent methylation of phosphatidylethanolamine to phosphatidylcholine by the SAM-dependent *phosphatidylethanolamine N-methyltrans-ferase* (PEMT), followed by the conversion to choline, then to betaine by several successive enzymatic activities (Sherriff et al., 2016) (Figure 1). The re-methylation of homocysteine to methionine using betaine is thus the salvage of methyl groups from phospholipids. Choline is an essential nutrient in mouse, human and many other vertebrates because it is a source of betaine as well as phosphatidylcholine.

### SAM-Binding MTases

As we have seen above, several enzymes use folates as 1-carbon donors resulting in the methylation of their substrate. However, virtually all methyltransferases use SAM as a cofactor. SAM-binding proteins are usually classified into 9 superfamilies based on domain structures (Martin and McMillan, 2002; Schubert et al., 2003; Petrossian and Clarke, 2009a, 2009b). In 2011, based on primary sequences, predicted secondary structures and solved crystal structures of known and putative *S*-adenosylme-thionine-dependent methyltransferases, human MTases were assigned to the 9 superfamilies of MTases (Petrossian and Clarke, 2011). It is estimated there are between 200 and 300 methyltransferases in the human genome (Petrossian and Clarke, 2011).

### Pathologies and Treatments Related to 1 C Metabolism

1 C metabolism is at the origin of many pathologies, and comprehensively covering this subject is out of the scope of this perspective. The reader is invited to consult selected previous works (Ducker and Rabinowitz, 2017; Carmel and Jacobsen, 2001; Lippi and Plebani, 2012; Longnecker, 2002; Poirier et al., 2003).

Due to its role in *de novo* dTMP and purines synthesis, the folate cycle is essential for rapidly proliferating cells and tissues, for which dietary folate is critical. The main dietary form of folate is normally polyglutamylated CH_3_-THF, in high amounts in green leafy vegetables (from Latin, *folium* means leaf). Natural folates are, however, highly unstable during food storage and preparation, resulting in folate deficiency to be relatively common. Folate deficiency was recognized as a frequent cause of anemia in children and adults in the early 1940s (Hoffbrand and Weir, 2001), and of neural tube defects in newborns in 1965 (Crider et al., 2011), prompting many governments to recommend fortification of flours and cereals with folic acid, a stable form of folate, in the late 1990s. Folate supplementation was also found to reduce the risk of congenital heart defects (Bailey and Berry, 2005).

Many inborn errors of 1 C metabolism have been identified in humans, including lethal AHCY mutations (Vugrek et al., 2009), MTR deficiencies causing a wide array of hematological and neurological deficits (Watkins and Rosenblatt, 1989), MTHFR and CBS polymorphisms causing hyperhomocysteinemia and homocystinuria associated with nervous, connective tissues and vascular damages (Schwahn and Rozen, 2001; Carmel and Jacobsen, 2001; Kraus et al., 1999; Kozich and Kraus, 1992), an MTHFD1 polymorphism (Brody et al., 2002) and MTHFD1 L variants associated with neural tube defects (Parle-McDermott et al., 2009) and late-onset Alzheimer’s disease (Naj et al., 2010). Very recently, an MTHFR polymorphism has been associated with chronic insomnia that could be resolved by targeting MTHFR’s loss of function (Kapoor et al., 2021).

Considering the importance of 1 C metabolism for proliferation, it is not a surprise that it is a major target for chemotherapies: methotrexate, permetrexed and their analogues are antifolates, inhibiting DHFR and other folate-dependent enzymes (Gonen and Assaraf, 2012); methotrexate is also recommended as the standard treatment for arthritis (Cronstein and Aune, 2020). Surprisingly, SAM has also been shown to block the growth of cancer cells (Ilisso et al., 2018; Mosca et al., 2020; Wang et al., 2017), and has been inconclusively proposed as a treatment for depression (Mischoulon et al., 2015), arthritis (Najm et al., 2004), and liver diseases (Guo et al., 2015).

Known 1 C pathologies are related to proliferative tissues because their symptoms are the most obvious, but work in mice have shown that 1 C metabolism deficiencies in adult animals have consequences in non-proliferative tissues. For example, knock-out of MTHFR, BHMT, CBS, or PEMT in mice all cause liver steatosis (Maclean et al., 2010; Chen et al., 2001; Schwahn et al., 2003; Teng et al., 2011; Zhu et al., 2003), which resonates with increased homocysteine often found in patients with non-alcoholic fatty liver disease (Dai et al., 2016).

While there is no question 1 C metabolism underlies many aspects of biology, this very essentiality—knock-out of MTHFD genes, AHCY, MTR, MAT2A are all lethal, and that of MTHFR is semi-lethal depending on the genetic background (Miller et al., 1994; Ducker and Rabinowitz, 2017; Swanson et al., 2001; Chen et al., 2001; Lawrance et al., 2011; Dickinson et al., 2017)—has hampered a clear understanding of its regulations and ramifications. Future investigations with modern genetic and metabolic tools should clarify the physiological functions of key enzymes of 1 C metabolism and how they are regulated in adults.

## Circadian Influence on Methyl Metabolism

### Links Between the Methyl Cycle and the Circadian Clock

Now that we have an overview of how C1 metabolism is organized, we can start looking into how it is linked with the clock. The rhythmic transcriptional programs underlying circadian rhythms in organisms from cyanobacteria to humans have been shown to be dependent on methylation (Fustin et al., 2020). Disruption of the methyl cycle with a pharmacological inhibitor of *Adenosylhomocysteinase* (AHCY, Figure 1) to induce accumulation of SAH, which can displace SAM from SAM-binding methyltransferases and act as a competitive inhibitor (Carmel and Jacobsen, 2001), led to the lengthening of the circadian period in all eukaryotic cells tested (Fustin et al., 2020). This shows that there is a link between circadian rhythms and methyl metabolism conserved during evolution. Available evidence also indicate that this link is bidirectional, *i.e.* that methyl metabolism as a whole is under the control of the circadian clock. Rhythms in histone (Koike et al., 2012), DNA (Oh et al., 2018) and mRNA (Zhong et al., 2018) methylation have been observed, and there has been suggestions that the methyl cycle and its outputs (glutathione and polyamine synthesis), may be under clock control, notably from the observations that polyamines, SAM and SAH display circadian oscillations in the liver (Eckel-Mahan et al., 2012; Atwood et al., 2011; Panda, 2016; Krishnaiah et al., 2017). Moreover, several MTases have been linked to circadian rhythms: the class V-like, SET domain-containing histone lysine MTases EZH2 (Etchegaray et al., 2006), KMT2A (Katada and Sassone-Corsi, 2010), KMT2 C (Valekunja et al., 2013) and KMT1/SUV39 H1 (Duong and Weitz, 2014), the class I-like mRNA *N*^6^-adenosine MTAse METTL3 and cap *N*^7^-guanine MTase RNMT (Fustin et al., 2013). More recently, AHCY was reported to directly interact with BMAL1 to support the histone methylation that accompanies rhythmic gene expression (Greco et al., 2020).

To provide a comprehensive review of the circadian influence on C1 metabolism at the transcriptional level, we appended the latest Gene Ontology (GO) source files and their annotations from geneontology.org with a new “*C1 metabolism*” (GO:9999999) biological process node containing all proteins shown in Figure 1. The modified GO source files are provided as supplemental information. Next, we re-analyzed the Gene Expression Omnibus dataset GSE54652, a high quality microarray-based transcriptomics atlas from various mouse tissues sampled at 2 h intervals for 2 days (Zhang et al., 2014), with the newest version of MetaCycle, an R package frequently used for detecting rhythmic signals from large scale time-series data (Wu et al., 2016). Genes whose expression was significantly rhythmic (Benjamini-Hochberg-adjusted *p* value < 0.05) in each tissue were identified, and GO analysis was performed using the appended GO source files on the Galaxy server (usegalaxy.org) using the GOEnrichment tool (Afgan et al., 2018). One GO analysis was performed on rhythmic genes for each tissue, and additional analyses were performed with genes that were rhythmic in at least a third of the tissues, and with all rhythmic genes in any tissues. To filter the most significant hits, only the biological processes enriched at least 2-fold and with a *q* value < 0.05 were considered. The MetaCycle and GOEnrichment outputs are provided in Supplementary Tables S1 and S2, respectively.

**Figure 2.**
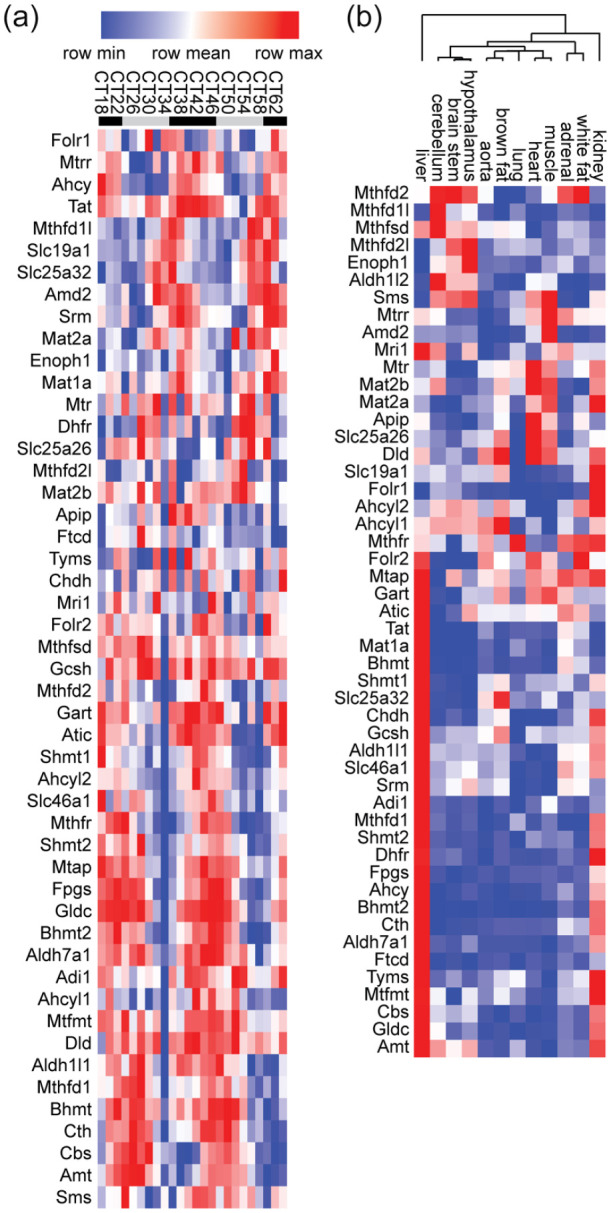
Orchestration of hepatic folate/methyl metabolism by the circadian clock. (a) Heatmap representation of expression values from GSE54652 of all genes shown in Figure 1, in the liver. Rows were organized by Pearson correlation-clustering using GenePattern (Reich et al., 2006; de Hoon et al., 2004; Eisen et al., 1998), leading to the grouping of genes with similar expression patterns. The legend at the top shows Circadian Time (CT), 0 (24, 48) being the start of the rest phase of the mouse. Sampling frequency was every 2 h, but for readability labels only show every 4 h. Mice were kept in constant darkness throughout the experiment; the start of the active phase in these conditions being determined by the endogenous circadian clock. Gray bars represent the inactive phase (“subjective” day), black bars the active phase (“subjective” night). Notice the difference in phase between genes at the top of the heatmap and toward the lower half. (b) Pearson correlation-clustering of mean gene expression in GSE54652 across 48 h of the same genes as in (a) in all tissues investigated. Notice the high expression of most genes in the liver, followed by the kidneys. The tree at the top shows the results of the Pearson correlation-clustering that was also performed on the columns. Notice the significant separation between expression patterns in brain tissues and peripheral tissues. See also supplemental information for raw expression values in all tissues.

Several observations stood out. First, *C1 metabolism* was the second most significant hit in the liver, immediately following the “circadian rhythms,” clearly showing the profoundly circadian organization of C1 metabolism in this tissue, where 31 out of 50 genes were significantly rhythmic (Figure 2a). Further indicating the liver as a major regulator of methyl metabolism, more than half of the *C1 metabolism* enzymes analyzed here were enriched in the liver (in terms of expression averaged across 48 h, Figure 2b). Second, *C1 metabolism* was significant in the hypothalamus, where the mammalian master clock resides (Moore and Eichler, 1972; Stephan and Zucker, 1972). Two related GO terms, *transsulfuration* and *sulfur amino acid biosynthetic process*, were also significantly enriched in this tissue. Third, *C1 metabolism* remained among significant hits when GO analysis was performed on genes rhythmic in at least 4 of the 12 tissues. And last but not least, when all rhythmic genes from any tissues were grouped for GO analysis, the most significant GO term was *C1 metabolism*, because out of the 50 genes included in *C1 metabolism*, 43 were found to be rhythmic in at least 1 tissue.

**Figure 3.**
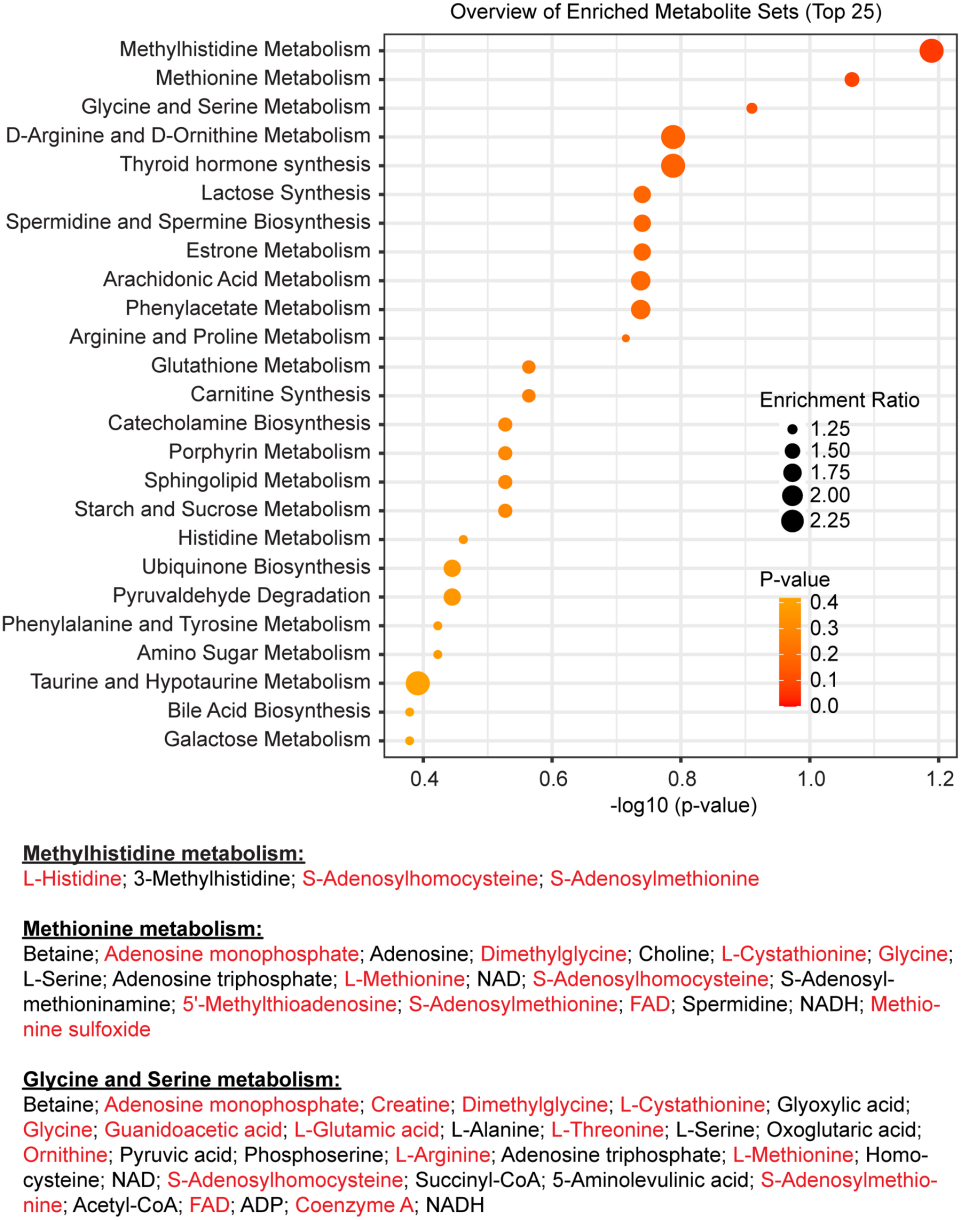
Orchestration of hepatic folate/methyl metabolism by the circadian clock. Results of overrepresentation analysis by Metaboanalyst 5.0 using previously published data (Krishnaiah et al., 2017). The most significant hits are all related to 1 C metabolism. At the bottom are listed all metabolites belonging to each of these three pathways present in the dataset, the rhythmic ones written in red. NAD = Nicotinamide adenine dinucleotide; FAD= Flavin adenine dinucleotide; NADH = Reduced nicotinamide adenine dinucleotide; ADP = adenosine diphosphate.

To further probe the link between 1 C metabolism and circadian rhythms, we next looked at the high temporal resolution metabolome profiling published by Krishnaiah and co-authors (Krishnaiah et al., 2017), and performed overrepresentation analysis using Metaboanalyst 5.0 (Pang et al., 2021; Xia et al., 2009), using rhythmic liver metabolites (BH.Q.< 0.01 in Dataset S2 from Krishnaiah et al.) as test dataset and all detected metabolites from the same table as reference (using The Small Molecule Pathway Database as a library (Jewison et al., 2014)]. The results, presented in Figure 3, lead to the same conclusion as above: the 3 most significantly enriched metabolic pathways were methylhistidine metabolism, methionine metabolism and glycine and serine metabolism, all overrepresented notably due to the fact that both SAM and SAH, at the crossroad of these pathways, are rhythmic, together with other C1-derived metabolites including methylthioadenosine (MTA), glycine, histidine and cystathionine (Figure 3).

Together these observations highlight the strong link between the circadian clock and C1 metabolism in mammals. The molecular mechanisms underlying this influence, and the physiological function of such a temporal organization, remain to be identified.

## Selected Further Questions

### What Is the Physiological Meaning of Temporal Organization?

**Figure 4.**
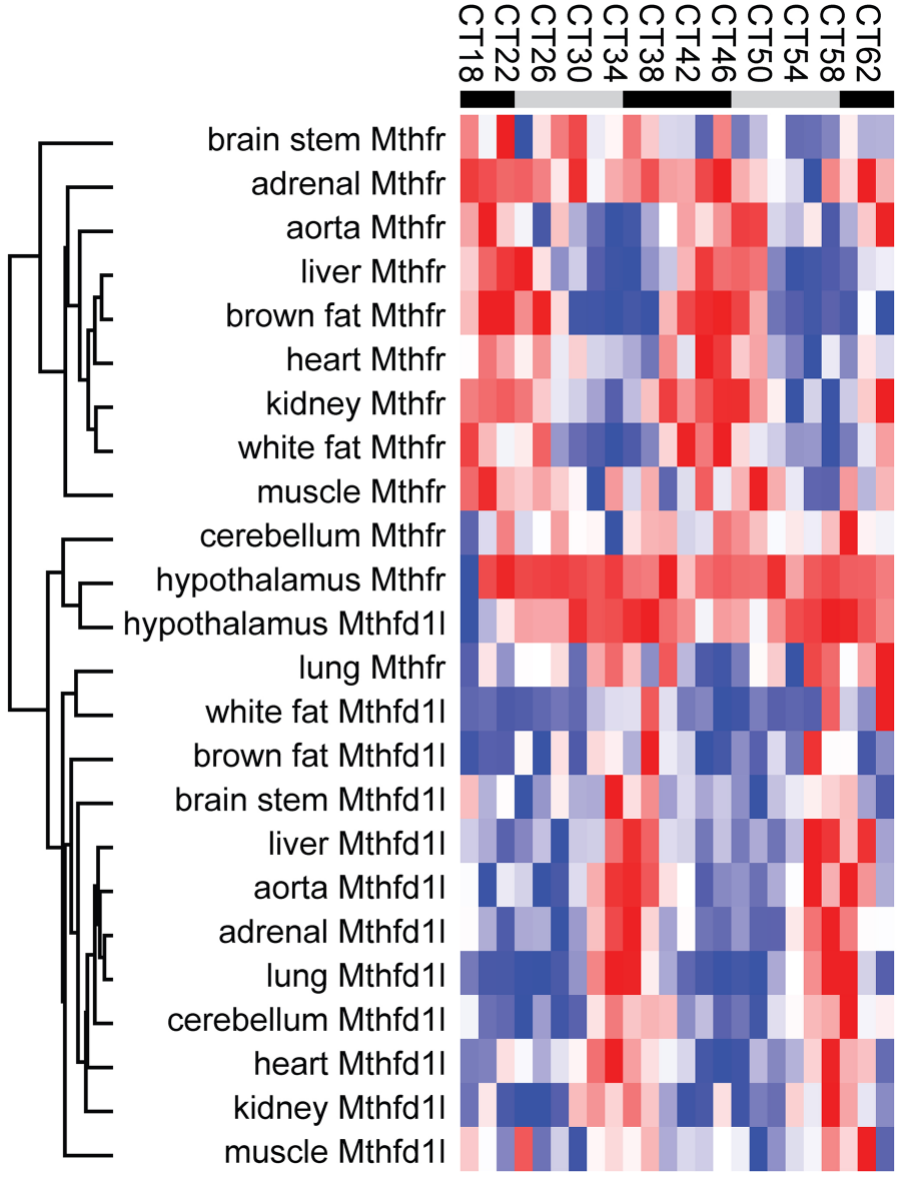
Expression of *Mthfd1l* and *Mthfr* in most tissues. Expression values for *Mthfd1l* and *Mthfr* in GSE54652 were clustered by Pearson correlation, leading to the clear separation of *Mthfd1l* and *Mthfr* into two groups with very few exceptions. Notice higher expression of both genes in the hypothalamus, at the center of the heatmap. Sampling frequency was every 2 h, but for readability labels only show every 4 h.

Among the few genes consistently rhythmic were *Mthfd1l* and *Mthfr*, their antiphasic expression (Figure 4) in many tissues especially interesting considering their key functions: MTHFD1 L provides formate to the cytoplasmic folate arc, and MTHFR is the critical link between cytoplasmic folate metabolism and the methyl cycle (Figure 1). This antiphasic expression prompts many questions. Is formate mainly released into the cytoplasm during the night (active phase in mice), activating cytoplasmic folate metabolism, when *Mthfd1l* expression is high? And is CH_3_-THF mainly produced during the day, when *Mthfr* expression is high? Why should it be so? Interestingly, hepatic SAM is highest during the subjective day, at CT4, further suggesting a link between the day and methylation (Krishnaiah et al., 2017). What would happen if the link between the circadian clock and folate/methyl metabolism is severed, for example by removing circadian rhythms of *Mthfr* or *Mthfd1l* expression?

### A Master for All, or a Division of Labor?

Based on average expression levels across 24 h of genes involved in methyl/folate pathways (Figure 4), there is a considerable level of tissue specificity, with the liver clearly a master regulator in mouse. *Ahcy* and *Mat1a* are enriched in the liver, while their respective homologues *Ahcyl1, Ahcyl2* and *Mat2a* are expressed ubiquitously at comparable levels. What is the contribution of each of these genes to systemic folate and methyl metabolism, and what is their involvement in the development of the numerous pathologies related to methylation deficiencies, including cancer, hepatitis, fibrotic and vascular diseases and neurological disorders?

### How Is Methyl Metabolism Regulated in Other Species?

The focus of this review was mammalian methyl metabolism, but what about other species? The circadian clock in invertebrates, plants, unicellular green algae and cyanobacteria is also sensitive to methylation inhibition (Fustin et al., 2020), but is the folate/methyl metabolism in these organisms also orchestrated by the circadian clock? Moreover, is the folate/methyl metabolism in unicellular green algae and cyanobacteria associated not only with the circadian clock but also with the light/dark cycle? Indeed, it would be predicted from the discussion about the prebiotic origin of methylation above that higher methylation in single-celled organisms directly responsive to light should be associated with the light phase. A hint that this may be the case, at least for mRNA methylation, has recently been shown in *Arabidopsis* (Wang et al., 2021). Understanding methyl metabolism in plants and algae may be relevant for biomass production.

## Conclusions

Light-induced prebiotic methylations may have influenced the origin and evolution of life to become the complex array of regulatory mechanisms it is today, and its hypothesized association with daylight may have been an early determinant in the evolution of circadian rhythms. In extant organisms, evidence indicate an intimate relationship between the circadian clock and methyl metabolism. Yet, because of the complexity of the folate/methyl metabolism and the sheer variety of methyltransferases, the regulation of methyl metabolism, and its impact on physiology and behavior, remain poorly understood. Future investigations into the relationship between methyl metabolism and circadian rhythms using state-of-the-art genetic editing and metabolic tracing should provide further insights. Given the clinical importance of folate and methionine metabolism, a more thorough understanding of these pathways is needed.

## Supplemental Material

sj-gaf-4-jbr-10.1177_07487304221083507 – Supplemental material for Methyl Metabolism and the Clock: An Ancient Story With New Perspectives Click here for additional data file. Supplemental material, sj-gaf-4-jbr-10.1177_07487304221083507 for Methyl Metabolism and the Clock: An Ancient Story With New Perspectives by Jean-Michel Fustin in Journal of Biological Rhythms

sj-obo-3-jbr-10.1177_07487304221083507 – Supplemental material for Methyl Metabolism and the Clock: An Ancient Story With New Perspectives Click here for additional data file. Supplemental material, sj-obo-3-jbr-10.1177_07487304221083507 for Methyl Metabolism and the Clock: An Ancient Story With New Perspectives by Jean-Michel Fustin in Journal of Biological Rhythms

sj-xlsx-1-jbr-10.1177_07487304221083507 – for Methyl Metabolism and the Clock: An Ancient Story With New Perspectives Click here for additional data file. sj-xlsx-1-jbr-10.1177_07487304221083507 for Methyl Metabolism and the Clock: An Ancient Story With New Perspectives by Jean-Michel Fustin in Journal of Biological Rhythms This article is distributed under the terms of the Creative Commons Attribution 4.0 License ( https://creativecommons.org/licenses/by/4.0/) which permits any use, reproduction and distribution of the work without further permission provided the original work is attributed as specified on the SAGE and Open Access pages ( https://us.sagepub.com/en-us/nam/open-access-at-sage).

sj-xlsx-2-jbr-10.1177_07487304221083507 – for Methyl Metabolism and the Clock: An Ancient Story With New Perspectives Click here for additional data file. sj-xlsx-2-jbr-10.1177_07487304221083507 for Methyl Metabolism and the Clock: An Ancient Story With New Perspectives by Jean-Michel Fustin in Journal of Biological Rhythms This article is distributed under the terms of the Creative Commons Attribution 4.0 License ( https://creativecommons.org/licenses/by/4.0/) which permits any use, reproduction and distribution of the work without further permission provided the original work is attributed as specified on the SAGE and Open Access pages ( https://us.sagepub.com/en-us/nam/open-access-at-sage).
